# Continuous Subcutaneous Apomorphine Infusion in Parkinson’s Disease: A Single-Center, Long-Term Follow-Up Study of the Causes for Discontinuation

**DOI:** 10.3390/jpm11060525

**Published:** 2021-06-08

**Authors:** Tove Henriksen, Harry Staines

**Affiliations:** 1 Department of Neurology, University Hospital of Bispebjerg, 2400 Copenhagen, Denmark; 2 Sigma Statistical Services, Balmullo KY16 0BD, UK; harry.j.staines@gmail.com

**Keywords:** advanced Parkinson’s disease, apomorphine, adherence

## Abstract

(1) Background: Subcutaneous apomorphine infusion (SCAI) is one of the three main treatment options for motor fluctuations in advanced Parkinson’s disease (PD). The adherence to SCAI is generally considered to be low due to adverse events and because it is perceived as a treatment option to be used for a limited period only. We evaluated the reasons for discontinuation of SCAI in relation to when patients stopped treatment. (2) Methods: We reviewed the medical records of PD patients treated with SCAI at a single center, capturing patient demographics and the reasons for cessation of SCAI. (3) Results: 101 patients were included in the analysis, with a median time on treatment of 6.34 years. The main reasons for stopping SCAI were adverse events, death, and dissatisfaction with treatment. In the first 6 years of treatment, the predominant side effects leading to discontinuation were somnolence and hallucinations. (4) Conclusions: We suggest that SCAI can be an effective long-term treatment option for advanced PD, but it requires careful patient selection, a high level of communication with the patient and carer, and rigorous monitoring of the effects of treatment and for any adverse events so they can be promptly managed.

## 1. Introduction

After even a few years of treatment with levodopa, a large proportion of PD patients will experience motor fluctuations [1] which, over time, can become intolerable and have a substantial impact on their quality of life [2]. Apomorphine is a dopamine D1 and D2 receptor agonist [3]. In the 1980s, the introduction of subcutaneous apomorphine infusion (SCAI) [4] offered the first option to reduce these motor complications. Since the introduction of SCAI, several open-label studies have confirmed its efficacy in managing motor fluctuations and OFF periods [5]. However, the lack of randomized controlled trials downgraded SCAI in evidence-based reviews of the treatment of advanced PD [6]. The TOLEDO study was the first multicenter, double-blind, randomized, placebo-controlled trial of the effect of SCAI [7]. In this study, the efficacy and safety of SCAI was compared to placebo in 107 patients with PD, with persistent motor fluctuations despite optimized oral or transdermal treatment. It confirmed what had previously been published in open-label studies: that SCAI resulted in a significant reduction in OFF time and a significant increase in ON time without troublesome dyskinesias. The role of SCAI in the treatment paradigm, however, has been debated. It has been suggested that, due to high drop-out rates, it is mainly a tool to bridge from per oral treatment to deep brain stimulation (DBS) or levodopa–carbidopa intestinal gel (LCIG) infusion [8,9], or as rescue medication in sudden OFF periods, while others regard it as a long-term treatment option to be compared with LCIG or DBS [10,11,12,13,14]. Table 1 summarizes the advantages and disadvantages of SCAI.

In this paper, we review data from a long-term follow up of 101 PD patients from our own Movement Disorder Clinic, focusing on adherence to SCAI and reasons for discontinuation, with an emphasis on timing. Our median time on treatment is longer than what has been published so far. Factors influencing adherence will be discussed, and with that, possible ways to improve it. 

## 2. Materials and Methods

### 2.1. Study Design

This was a single-center, observational study using the medical records of all patients who were diagnosed with PD according to the UK Parkinson’s Disease Society Brain Bank criteria [15] and started treatment with SCAI from 2002 until end of 2014. The reasons for starting the treatment, and, if appropriate, the cause for discontinuation were noted. In most cases, the level of satisfaction with the treatment was noted from both the patient’s and the treating neurologist’s perspective. 

### 2.2. Ethics

Ethical review and approval were waived for this study by the local Ethical Committee, because it considered the study to be a quality assurance measure and, as a result, informed consent was not required from the patients.

### 2.3. Outpatient Clinic Set-Up

In our Movement Disorders Center, patients were followed from when they started SCAI therapy and throughout the study inclusion period. It is a tertiary center to which patients are referred with advanced PD, namely those PD patients who are not adequately controlled with per oral or transdermal medication. This definition has not changed over time, however based on our experience with the effect of SCAI, we no longer offer it to patients with anterocollis. Patients are offered either DBS (since 1999), SCAI therapy (since 2001) and LCIG infusion (since 2004). Over the period of this study, patients were treated primarily by four movement disorders (MDS) specialists. A single PD specialist nurse managed all patients being treated with SCAI throughout the study period (except for one year). The treatment selection was carried out by the four MDS using available data on clinical effect and possible adverse effects and our shared knowledge about device-aided treatments. Some patients were regarded as good candidates for more than one of the treatment options. In those cases, we made a shared decision with the patient and carer. The age limit for DBS was approximately 70 years and patients with some cognitive problems were excluded from DBS treatment. From the beginning, we were reluctant to offer SCAI to patients with bothersome orthostatic hypotension or a history of severe psychosis, and even more so after the introduction of LCIG. Patients with prior abdominal surgery were not considered good candidates for LCIG due to potential difficulties placing the PEG tube. Patients with a reduced life expectancy were good candidates for SCAI as it does not entail surgery. Until 2004, approximately 75% of the patients received treatment with DBS. After the introduction of LCIG in 2004, 50% received DBS, 25% received SCAI, and 25% received LCIG. Apomorphine injections are available at our center as rescue medication for sudden and unpredictable OFF episodes. 

### 2.4. Evaluation and Treatment of Advanced PD Patients

All candidates referred for advanced therapy were evaluated at monthly meetings with the four MDS, the PD nurse specialist, and two neuropsychologists. All patients had dopamine transporter single photon emission tomography performed and a computed tomography scan of the brain and/or magnetic resonance imaging of the brain to confirm the diagnosis of PD. A neuropsychological work-up was performed to evaluate cognitive status and, in some patients, an additional evaluation by a psychiatrist was undertaken when there was suspicion of a problematic psychiatric disorder, such as severe depression with suicidal ideation. 

The patients had an apomorphine test and a levodopa challenge performed as an in-patient. In almost all patients, SCAI therapy was started in hospital during a 1–2-week admission period. Each patient was followed by the same PD nurse specialist, and in most cases the same MDS, throughout the period. Ultrasound physiotherapy was initiated from the beginning of SCAI to prevent the formation of noduli. During ultrasound physiotherapy 0.5 Watt/cm^2^ was applied for 3 min per nodule or at each injection site by a trained physiotherapist, at home or at the clinic. The ultrasound head was moved slowly in circles during the treatment. Patients never applied the treatment themselves due to the risk of skin burns. In most patients, this treatment was stopped within a few weeks; in others, the frequency was reduced to twice per month. In some cases, it was continued on a weekly or biweekly basis, but for most patients this was not necessary. 

The needles used for SCAI infusion were either Neria with a 90-degree angle or Neria with a 45-degree angle. For a short period, the CLEO system was used but results were disappointing. Later we changed to using Neria Guard. If skin reactions occurred, we changed the length of needle, (lengths of 5 mm, 8 mm, 9 mm and 12 mm are available). Shorter needles were used in patients with low bodyweight and hence thin abdominal subcutaneous fat.

## 3. Results

A total of 101 patients who started SCAI treatment at our center, including those who were very early drop-outs, were included in the analysis. Indications for starting SCAI were: severe motor fluctuations (*n* = 75), painful off dystonia (*n* = 13), no effect of other PD medications (*n* = 8), severe tremor (*n* = 4), psychosis related to motor fluctuations (*n* = 4), severe off dysphoria (*n* = 3) and severe antecollis (*n* = 1). No patients received SCAI monotherapy or a 24-h infusion regimen.

**Figure 1:**
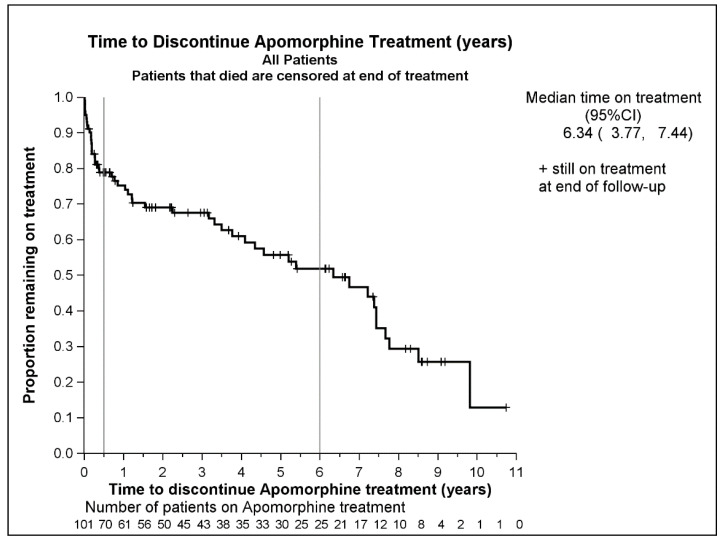
A Kaplan–Meier plot of time to discontinuation of SCAI. Discontinuation due to death was censored.

A Kaplan–Meier plot shows the time to discontinuation of SCAI treatment for all 101 patients (Figure 1). Overall, 69 patients (68%) discontinued SCAI treatment, with a median time on treatment of 6.34 years (95% CI: 3.77, 7.44). Adjusting for a variety of variables, only the patient assessment of satisfaction with treatment was a significant risk factor for discontinuation at the 5% significance level. The patient assessment was based on patient feedback noted by the neurologist or nurse in the patient’s medical records. A multivariable Cox Regression model was fitted showing that neither age (*p* = 0.64) nor gender (*p* = 0.14) was a significant risk factor for discontinuation, at the 5% level.

**Figure 2:**
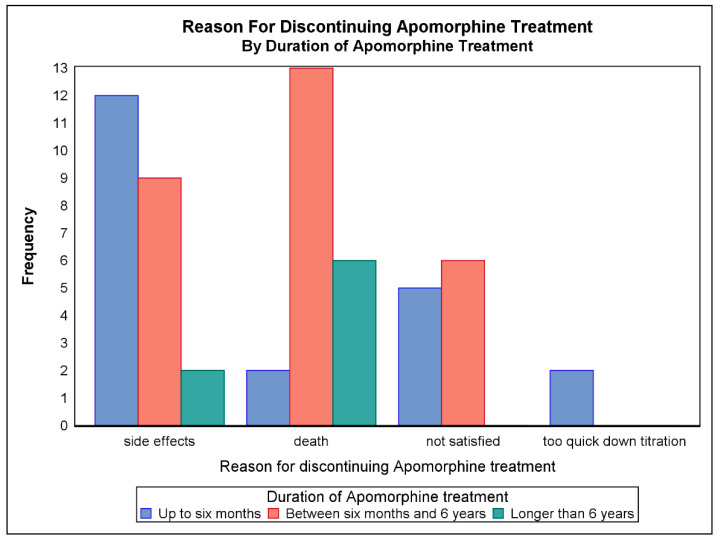
Reasons for discontinuing SCAI therapy according to duration of treatment.

Patients who discontinued were stratified into three groups according to the time at which they discontinued treatment: Group 1: up to 6 months after initiation; Group 2: from 6 months to 6 years; Group 3: after more than 6 years, and the reasons for discontinuing treatment were noted (Figure 2). A total of 31 patients (30.1%) discontinued SCAI treatment in the first 6 months and an additional 45 patients (44.6%) after 6 years. Of those who discontinued within 6 months, 63% said they were not satisfied with the treatment, in comparison to 30.0% and 11.5% in the periods 6 months to 6 years and over 6 years, respectively.

**Figure 3:**
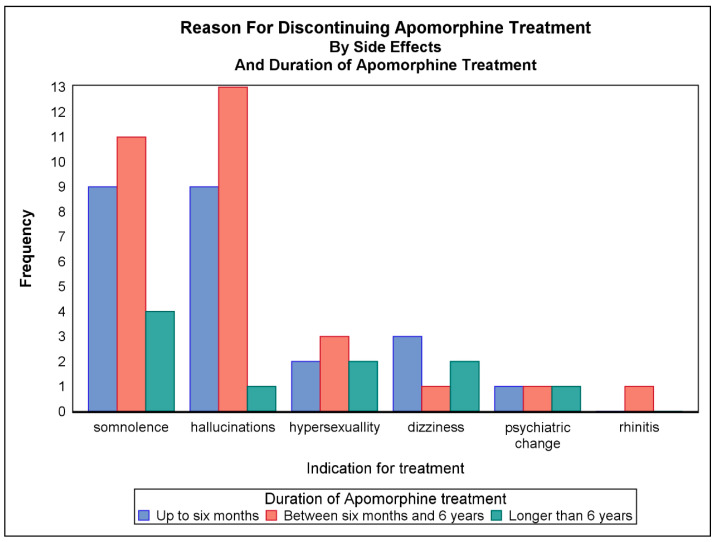
Adverse effects leading to discontinuation of SCAI therapy according to duration of treatment. Patients may have discontinued due to more than one adverse effect.

Figure 3 shows which adverse effects led to discontinuation of therapy according to the duration of treatment. Overall, the most common adverse effects that led to discontinuation were somnolence and hallucinations.

## 4. Discussion

Treatment adherence in general is a well-known challenge in PD [6]. It is related to lack of social support, depression, the number of PD medications that often need to be taken, and adverse events [16]. Device-aided therapies such as SCAI, DBS and LCIG should ideally reduce the complexity of the medical treatment of PD. However, an additional factor needs to be considered: the patient’s satisfaction with their treatment. A study of DBS therapy showed that 25% of patients were disappointed with the outcome [17]. In some cases, this was because the patients expected effects on axial symptoms [18], which DBS is less likely to reduce. In other cases, it was because they looked for effects relating to their professional life, interpersonal relationships, and leisure activities. This was especially the case in patients who had a more formal education [19]. 

SCAI is generally considered to be a treatment that is easy to start, but also easy to stop compared to LCIG or DBS, which may partly explain the high drop-out rates reported in the literature. In this study, we aimed to investigate reasons why patients discontinue SCAI therapy, factors that might influence adherence, and ways to improve this.

Previous studies of SCAI therapy report that the duration of continuation with SCAI treatment varies from 5.50 (SD, 3.8) months to 4.3 years (Table 2). This wide range reflects the different follow-up periods of individual studies which makes it difficult to draw direct comparisons between them. Patients included in this analysis had a median duration of treatment of 6.34 years, which is longer than reported in previously published data, and is partly due to our long follow-up period.

When comparing the causes for, and factors that influence, SCAI treatment cessation, of special interest is a retrospective study investigating adherence to SCAI treatment in two different settings: one center in Bangkok and one in Madrid [20]. The patients in the Thai cohort had a follow-up period of 27 (SD, 17.6) months (reflecting the limited time that SCAI infusion had been available), and the Spanish cohort 88.8 (SD, 96.2) months. A total of 52.7% of Thai patients discontinued compared with 62.5% of Spanish patients. In both cohorts, this mostly occurred within the first 6 months of starting treatment. There were, however, notable differences in the reasons stated for stopping treatment. In the Thai cohort, the main reason was skin nodules (36.8%), perceived lack of efficacy (15.8%), and hallucinations (15.8%). In the Spanish group, the reasons given were perceived lack of efficacy (43.8%), nausea (6.3%), and hemolytic anemia (6.3%), but interestingly there were no cases of psychosis [20]. Common reasons cited for discontinuation in other published studies include worsening of therapeutic effect, lack of improvement in dyskinesia, adverse effects, lack of motivation, cognitive impairment, lack of support, and depression/anxiety [7,8,9,10,11,12,13,14,20,21,22,23].

The specific adverse effects that are reported to lead to discontinuation are often skin reactions, psychosis, hypotension, and gastrointestinal complications. In the 12-week, double-blind period of the TOLEDO study, adverse events led to study withdrawal in 6 out of 53 patients in the active arm: 1 due to severe hypotension; 1 myocardial infarction, which was considered not to be related to the SCAI; 1 persistent moderate abnormal hematology; 1 due to visual hallucination; 1 moderate gait disturbance; 1 to mild infusion site erythema [7]. However, in a Spanish multicenter study, none of the 82 patients, who had a mean follow-up period of 19.93 ± 16.3 months, stopped SCAI treatment due to adverse effects [11]. 

Other, less tangible, factors can also influence whether patients continue with SCAI therapy. The Bhidayasiri et al. study observed that Thai patients were more prone to stop treatment if they developed skin nodules, whereas no patients in the Spanish group dropped out for this reason [20]. The authors note that SCAI is considered by Thai patients to be a ‘last resort’ treatment, and that dissatisfaction can increase if the patient experiences skin nodules [20], so cultural factors can have an influence on treatment adherence.

In a study by Colzi et al. [21] where patients had a mean of 4.5 years of SCAI treatment, there were reports of small cutaneous abdominal nodules, but none led to treatment cessation. This center used abdominal ultrasound physiotherapy, in the same manner as our center, suggesting that these skin problems can be manageable with a proactive approach. We started abdominal ultrasound physiotherapy from the beginning of SCAI treatment, and this could have had an important preventive effect of the formation of noduli and thereby increased adherence. The PD nurse specialist was careful to inspect the patient’s abdominal skin at every outpatient visit.

Opinion on the risk of developing hallucinations or psychosis with SCAI is somewhat mixed. Borgemeester et al. [24] found that the occurrence of visual hallucinations was reduced from 49% to 30% in patients on long-term SCAI [23].Manson et al. found that neuropsychiatric symptoms in general improved with SCAI therapy, especially in the group receiving SCAI monotherapy [10]. Ellis et al. mentioned that SCAI led to abolition or reduction of neuropsychiatric complications in all patients [25]. 

In our study, comparing discontinuation in the three subgroups, the reasons for cessation differed across these timepoints. In the first 6 months after initiation, 30% of patients discontinued SCAI treatment and, of these, 63% reported this was due to dissatisfaction with treatment. Death was a major reason after the first 6 months. Adverse effects and dissatisfaction with treatment were the main reasons for dropping out within the first 6 years. Down-titrating per oral medication too rapidly was only a problem in the first 6 months. Due to these drop-outs, we have subsequently changed the way we down-titrate, doing it at a slower pace.

The predominant side effects observed in the first and second time periods were hallucinations and somnolence. Adverse effects rarely led to discontinuation after 6 years of treatment. For some of the patients for whom the cause of cessation was recorded as ‘lack of satisfaction’, this term also covered reduced compliance or lack of support from the carer. Eleven patients stopped the treatment due to dissatisfaction. This was due to non-compliance of the carer in three cases (one case in the group that discontinued after 6 months and two cases in the group that discontinued between 6 months and 6 years). One patient in the first time-period group and one in the second group were also non-compliant with treatment. Of these patients, the majority were dissatisfied with the effect of treatment rather than the way it was administered. No patients in our study stopped treatment due to formation of skin nodules. In a retrospective study by Sesar et al. of 230 PD patients over 10 years, the longest duration of SCAI treatment was 124 months [14]. They found that the main reasons for discontinuation in the first year were adverse effects in 34.1%, patient decision in 19.5%, death in 7.3%, lack of family support in 7.3%, and what they termed ‘selection failure’ in 4.9%, which align with our overall findings. For some patients in this study, we used SCAI as a bridge to DBS. For one patient, SCAI turned out to be an effective solution, and he later refrained from having DBS performed.

The authors of several studies of SCAI therapy comment on the importance of full commitment to the treatment by the patient, the carer and the treating neurologist [11,22]. Our dedicated, single PD nurse specialist managing the SCAI patients could have been a contributing factor to our relatively low discontinuation rate. This nurse was on leave for one year over the study period. Usually, approximately 10 patients start SCAI each year at our center, but the year she was not on the team, no patients started SCAI treatment, although it is worth noting that there was not an excessive number of drop-outs. This highlights the importance of full engagement of the healthcare team in ensuring treatment uptake and success. Based on our own experience with SCAI, we have now prolonged the down-titration phase for oral medications. We have also recruited another PD nurse specialist, so it is easier if one is absent. We continue to use ultrasound physiotherapy and pay close attention to formation of noduli.

Alongside this, efforts also need to be made to give the patient and their family a realistic idea of what to expect in term of treatment effect. Rossi et al. suggest that physicians should help families better understand potential changes in family roles before surgery is performed [17]. A study showed, that patients who did not experience improvement of the symptoms they were most concerned about, underestimated the improvement of the treatment [27]. Involving the patient and carer in decision-making is equally important. Involvement correlates with patient satisfaction [28] and dissatisfaction with the communication in the outpatient clinic can result in poor compliance [29]. In a Spanish study of LCIG, 38% of patients stopped treatment, the majority within the first 3 months, and it was related to a perceived lack of efficacy of SCAI treatment or lack of acceptance of the device [30].

## 5. Conclusions

In this analysis of 101 PD patients at our center, the median time on SCAI treatment was 6.34 years, which is longer than has been reported previously. The results of our study confirm those of several others in the literature showing that patient dissatisfaction with treatment is commonly cited as a reason for treatment discontinuation. In order to change the perception of SCAI as being solely a bridge from per oral therapy to LCIG or DBS and for it to be viewed as a valid alternative to these treatments, we need to increase patient satisfaction with what can be an effective therapy for many.

The patient and carer should be well informed about the expected clinical effects of SCAI treatment, the adverse events to expect and the follow-up procedure. Even more so, we need to set realistic expectations and make clear what not to expect of the treatment. The patient should participate in the decision making. Close monitoring of the patient is advised, where an evaluation of any necessary adjustments to SCAI and per oral treatment can be performed. Experiencing adverse events does not mean that treatment has to be stopped in all cases. Most can be managed and SCAI can be continued. This is why it is important to be vigilant about monitoring adverse events and treating them promptly.

Hallucinations, for example, may be managed with cholinesterase inhibitor and clozapine to avoid unnecessary discontinuation of SCAI [24], and every effort should be made to employ good skin hygiene and management techniques to prevent the troublesome formation of skin nodules. The importance of a skilled and dedicated PD nurse is underpinned by observations at our center. Our SCAI-dedicated PD nurse was on leave for one year of the study period. Approximately 10 patients started SCAI each year at our center, but the year she was not on the team, no patients started SCAI treatment.

We suggest that SCAI can be an effective long-term treatment option for advanced PD, but it requires careful patient selection, a high level of communication with the patient and carer, and rigorous monitoring of the effects of treatment and for any adverse events so they can be promptly managed.

## Figures and Tables

**Table 1:** Advantages and disadvantages of SCAI as a treatment option for advanced PD.

Advantages	Easy to start	No surgery required	Possibility of using a bolus dose	Easy to stop	Small pump
Disadvantages	Not generally given as monotherapy	Requires good dexterity	Risk of adverse events	Easy to stop	Requires close follow-up

**Table 2:** Comparison of SCAI treatment duration and discontinuation rates from published studies.

Study	Patients (*n*)	Duration Of Treatment/Study Follow-Up	Discontinuation Rate (%)	Reasons for Discontinuation (*n*)
Colzi A, et al. (1998) [21] (Retrospective analysis)	10	Mean treatment duration: 4.5 years (range: 2.1–8)	Not reported	Not reported
Manson AJ, et al. (2002) [10] (Retrospective analysis)	64	Mean follow-up: 33.8 months (range: 2.1–8)	3 patients (4.7%) stopped within 6 months; a further 7 (10.9%) after long-term therapy	Within 6 months: difficulty with compliance; AEs such as daytime somnolence, skin complications, and painful dystonias. Longer-term: hemolytic anemia (1); behavioral problems (2); logistics (2), patient preference despite treatment efficacy (2)
García Ruiz PJ, et al. (2008) [10] (Retrospective analysis)	82	Mean (± SD) follow-up: 19.93 ± 16.3 months; 27 patients had received SCAI for >2 years and 9 for ≥4 years	Not reported	Not reported
Borgemeester RWK and van Laar T (2017) [24] (Retrospective analysis)	45	Follow-up: median 26 months	29 (64%; includes 17 patients who died during the study period after successful treatment for a median of 30 months); 6 patients withdrew after a median of 2 months; 4 patients discontinued after 9 months	After 2 months: due to AEs: orthostatic hypotension (3), visual hallucinations (1), excessive daytime sleepiness (1) and nausea (1) After 9 months: due to lessening of therapeutic effect
Sesar, Á, et al. (2017) [14] (Retrospective analysis)	230	Mean treatment duration: 26.3 months	137 (59.6%)	Adverse effects, psychosis being the most common
Katzenschlager R, et al. (2018) [7] (Randomized, double-blind trial)	107	12 week double-blind, placebo-controlled study	35 (32.7%) discontinued before the end of the double-blind phase (12 in the SCAI group, 23 in the placebo group)	SCAI group: adverse events Placebo group: lack of efficacy
Bhidayasiri R, et al. (2019) [20] (Retrospective analysis)	36 (Thai cohort); 16 (Spanish cohort)	Mean (± SD) follow-up: 27 (± 17.6) months (Thai cohort) and 88.83 (± 96.2) months (Spanish cohort)	19 (52.7%) in the Thai cohort and 10 (62.5%) in the Spanish cohort discontinued within approximately 6 months of initiation	Thai cohort: Skin nodules (7) perceived lack of efficacy (3), hallucinations (3), dyskinesia (2), hypotension (1), difficulty with device (1), other reasons (2) Spanish cohort: Perceived lack of efficacy (7), insufficient dexterity to handle device (1), nausea (1), hemolytic anemia (1), other reason (1)
Olivola E, et al. (2019) [8] (Retrospective analysis)	114	Patients treated with SCAI for at least 6 months over the study period (January 1998 to December 2012)	Discontinuation of SCAI therapy was an inclusion criterion of the study	Most common reason for discontinuation was lack of dyskinesia improvement (36.8%); second most common reason was cognitive deterioration
Katzenschlager R, et al (2021) [26] (52-week open-label phase of a randomized, double-blind trial)	84	Median duration of Treatment: 52.1 weeks (quartile 1: 32.8, quartile 3: 53.1).	25 (29.8%)	14 of the 25 patients who discontinued did so due to adverse events; only infusion site reactions (4) and fatigue (2) occurred in more than one patient.

## Data Availability

Not applicable.
